# Supplementary figures and images for: Repurposing the mammalian RNA-binding protein Musashi-1 as an allosteric translation repressor in bacteria
Source: eLife. 2024 Feb 16;12:RP91777. doi: 10.7554/eLife.91777 (PMC10942595; doi:10.7554/eLife.91777)

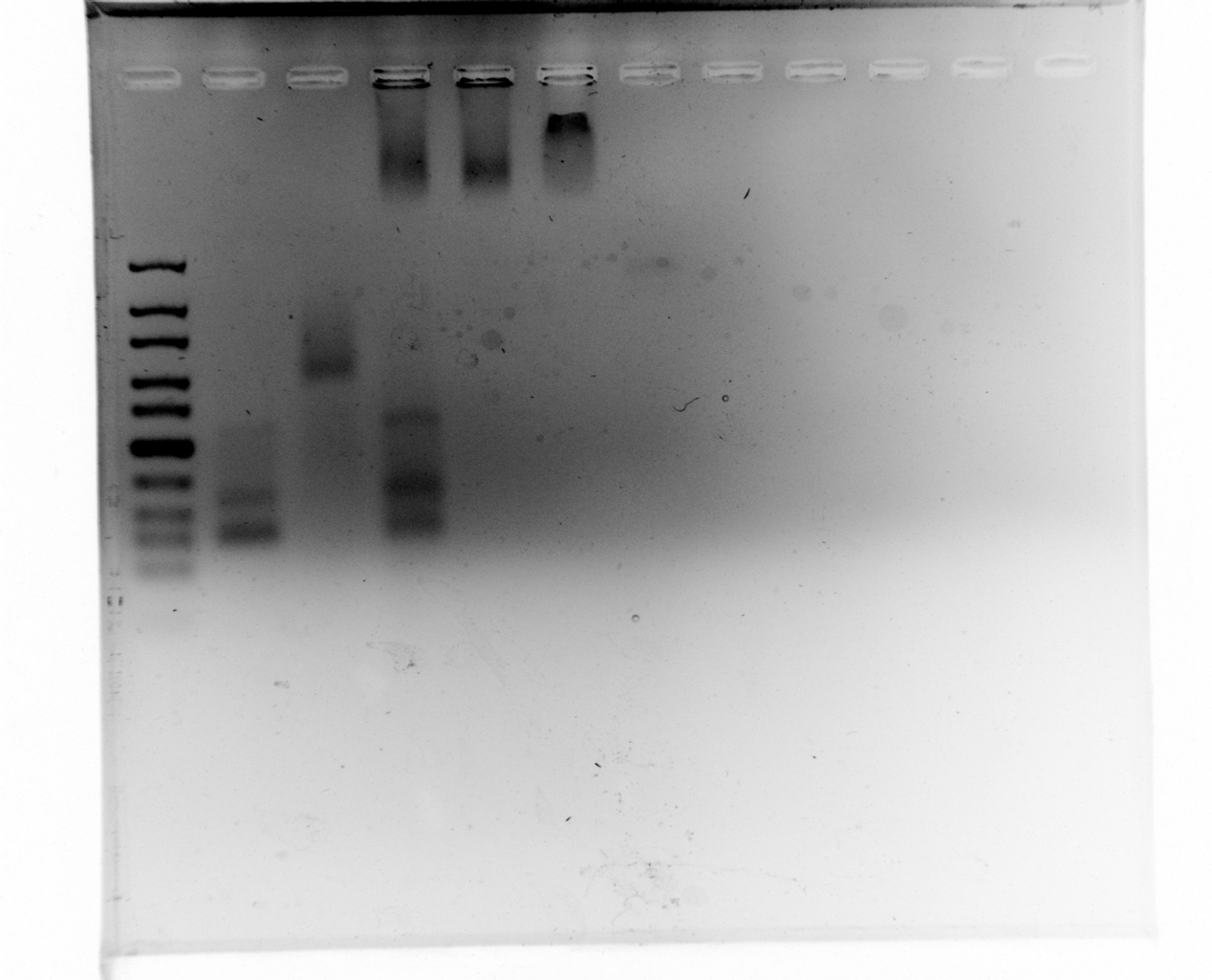

Supplement: Figure 5—source data 2. [file elife-91777-fig5-data2.zip › gel_fig5bLeft.png]

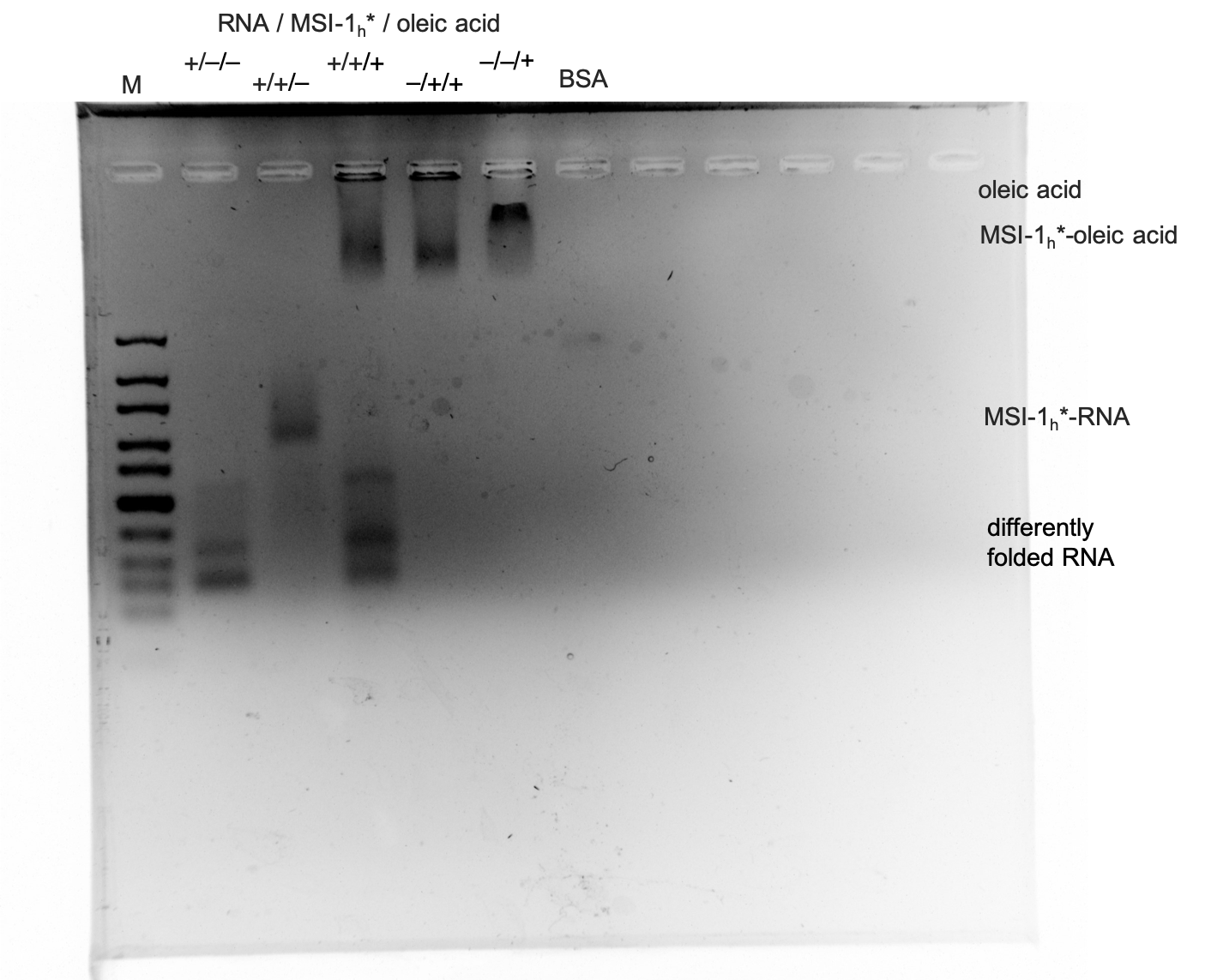

Supplement: Figure 5—source data 2. [file elife-91777-fig5-data2.zip › gel_fig5bLeft_labels.png]

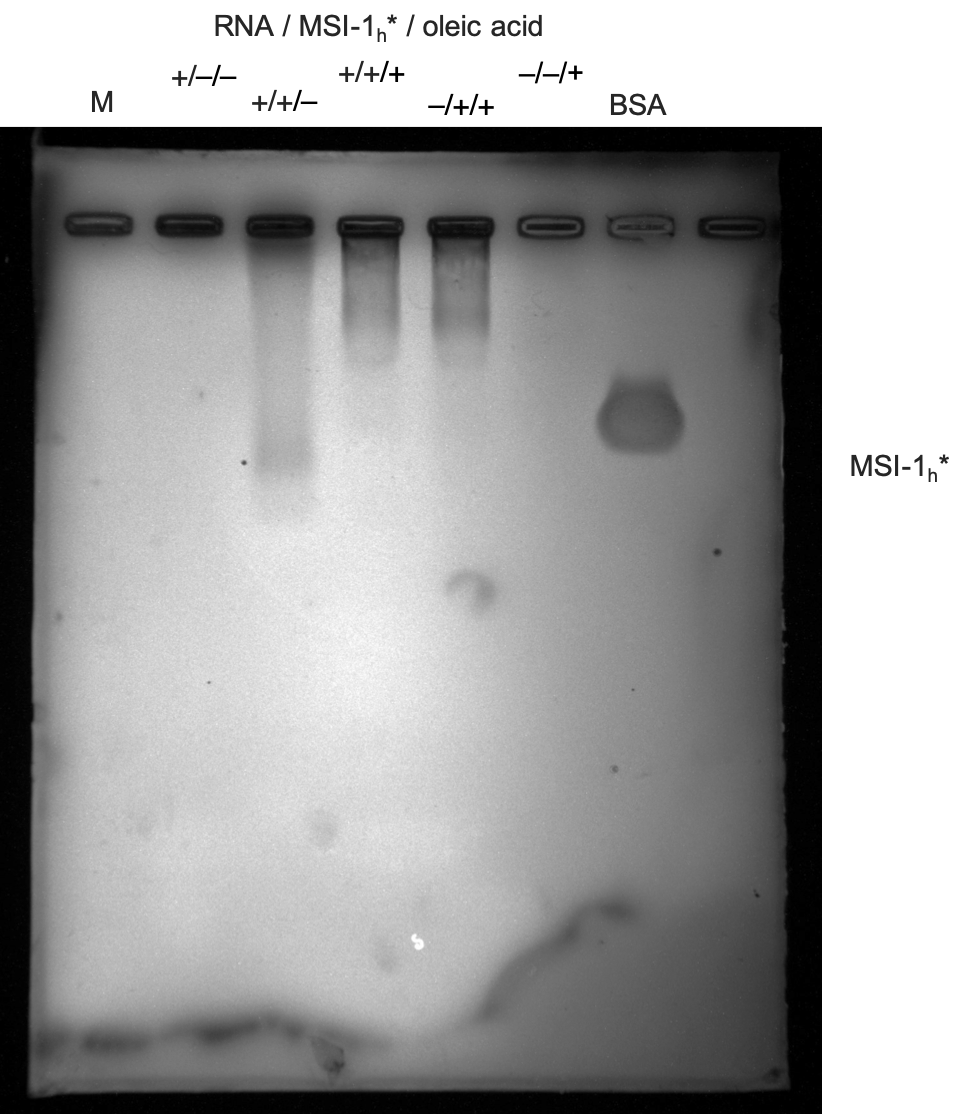

Supplement: Figure 5—source data 2. [file elife-91777-fig5-data2.zip › gel_fig5bRight_labels.png]

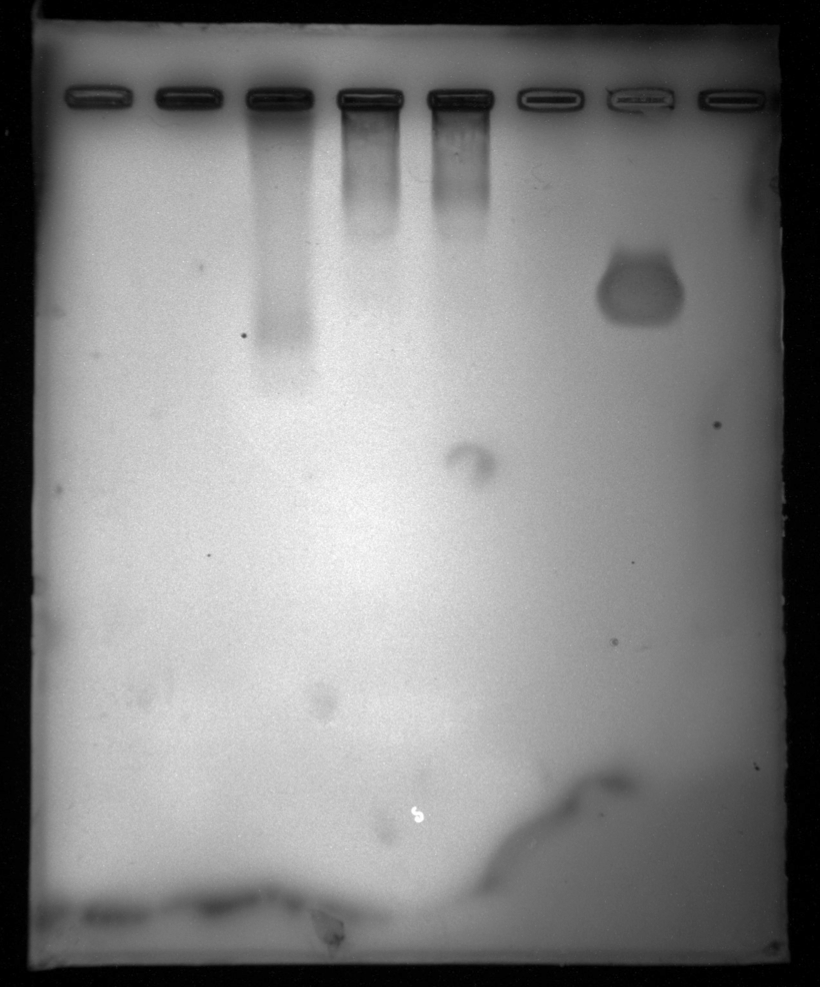

Supplement: Figure 5—source data 2. [file elife-91777-fig5-data2.zip › gel_fig5bRight.png]

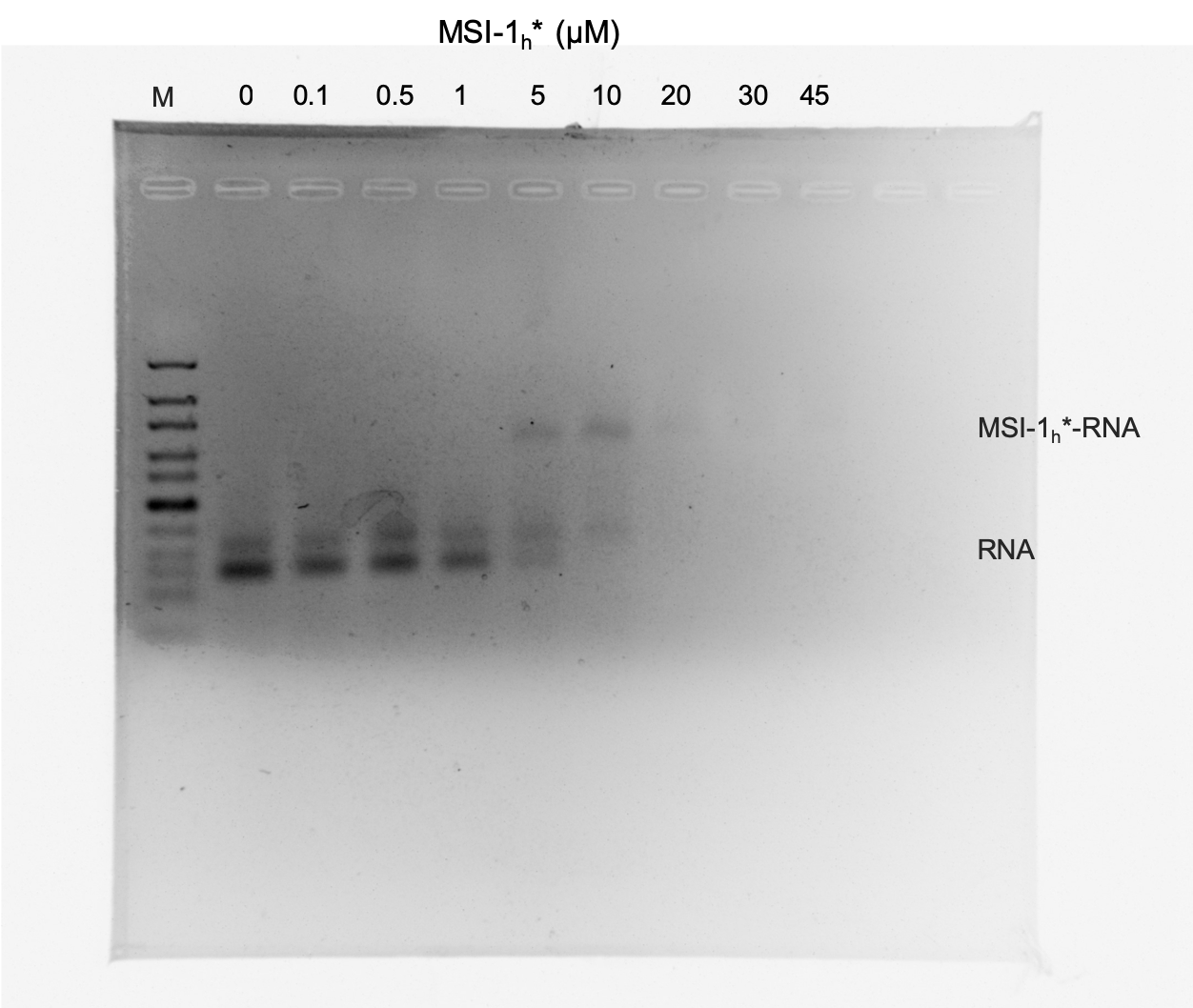

Supplement: Figure 5—figure supplement 1—source data 1. [file elife-91777-fig5-figsupp1-data1.zip › gel_fig5-figsuppl1a_labels.png]

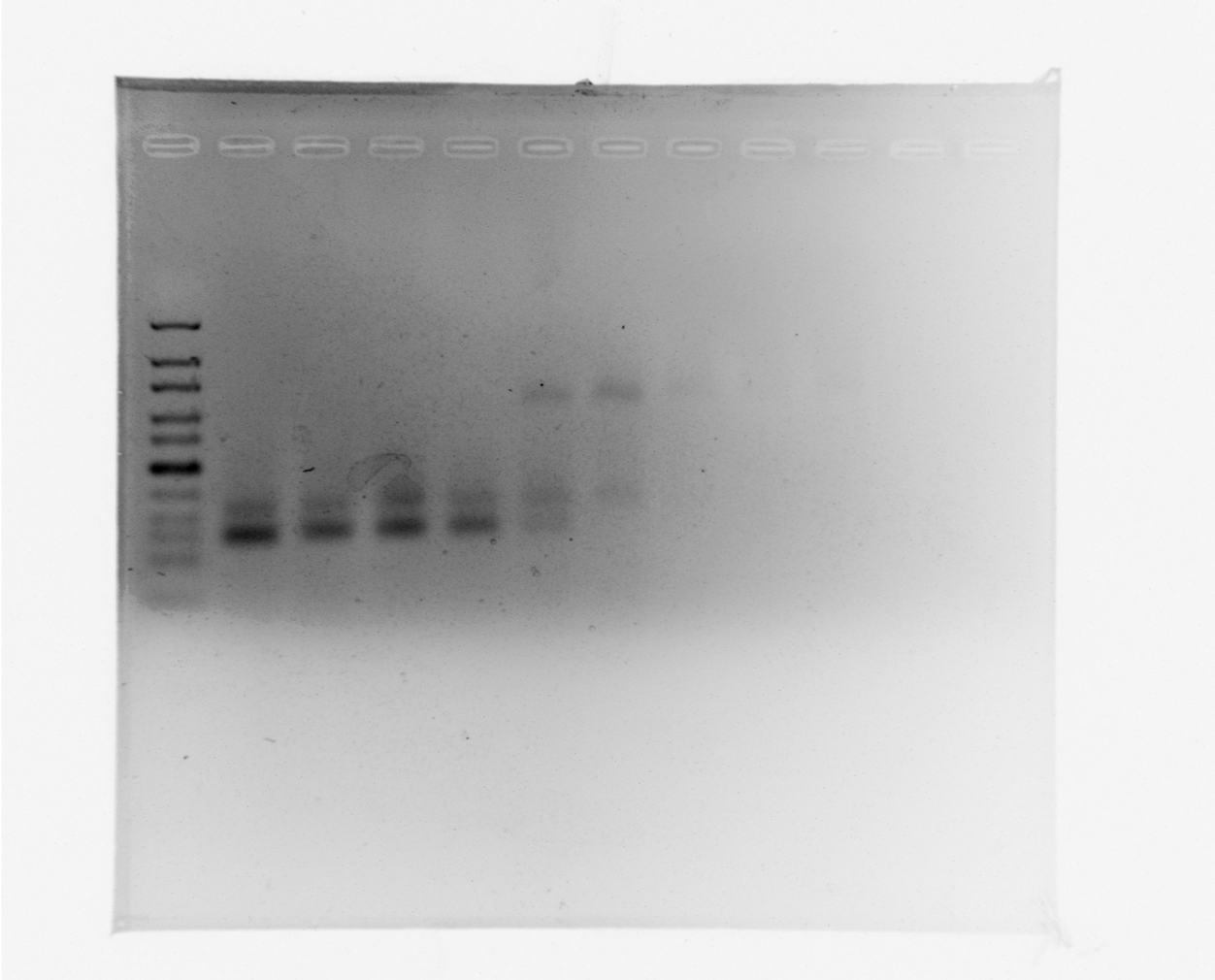

Supplement: Figure 5—figure supplement 1—source data 1. [file elife-91777-fig5-figsupp1-data1.zip › gel_fig5-figsuppl1a.png]

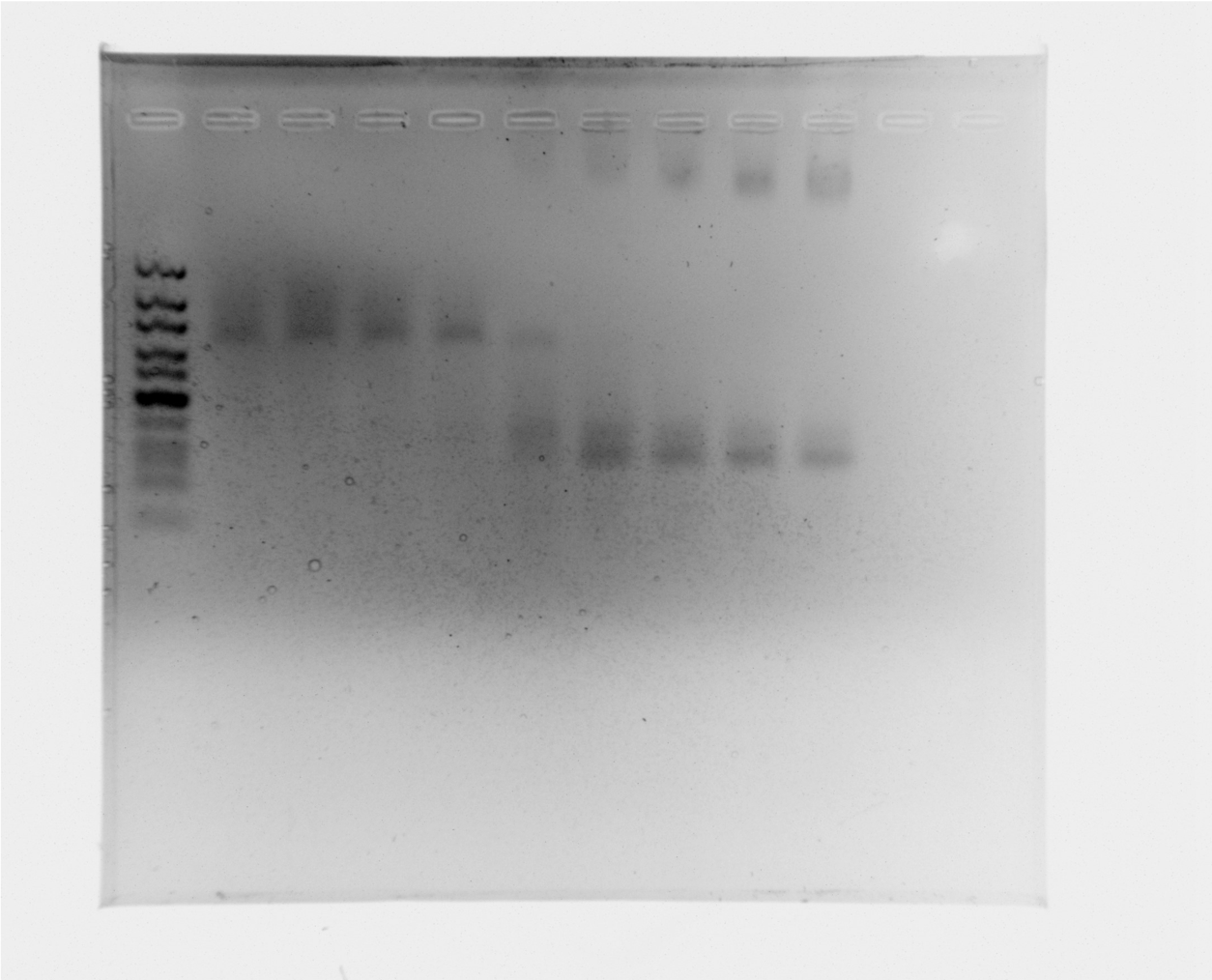

Supplement: Figure 5—figure supplement 1—source data 1. [file elife-91777-fig5-figsupp1-data1.zip › gel_fig5-figsuppl1b.png]

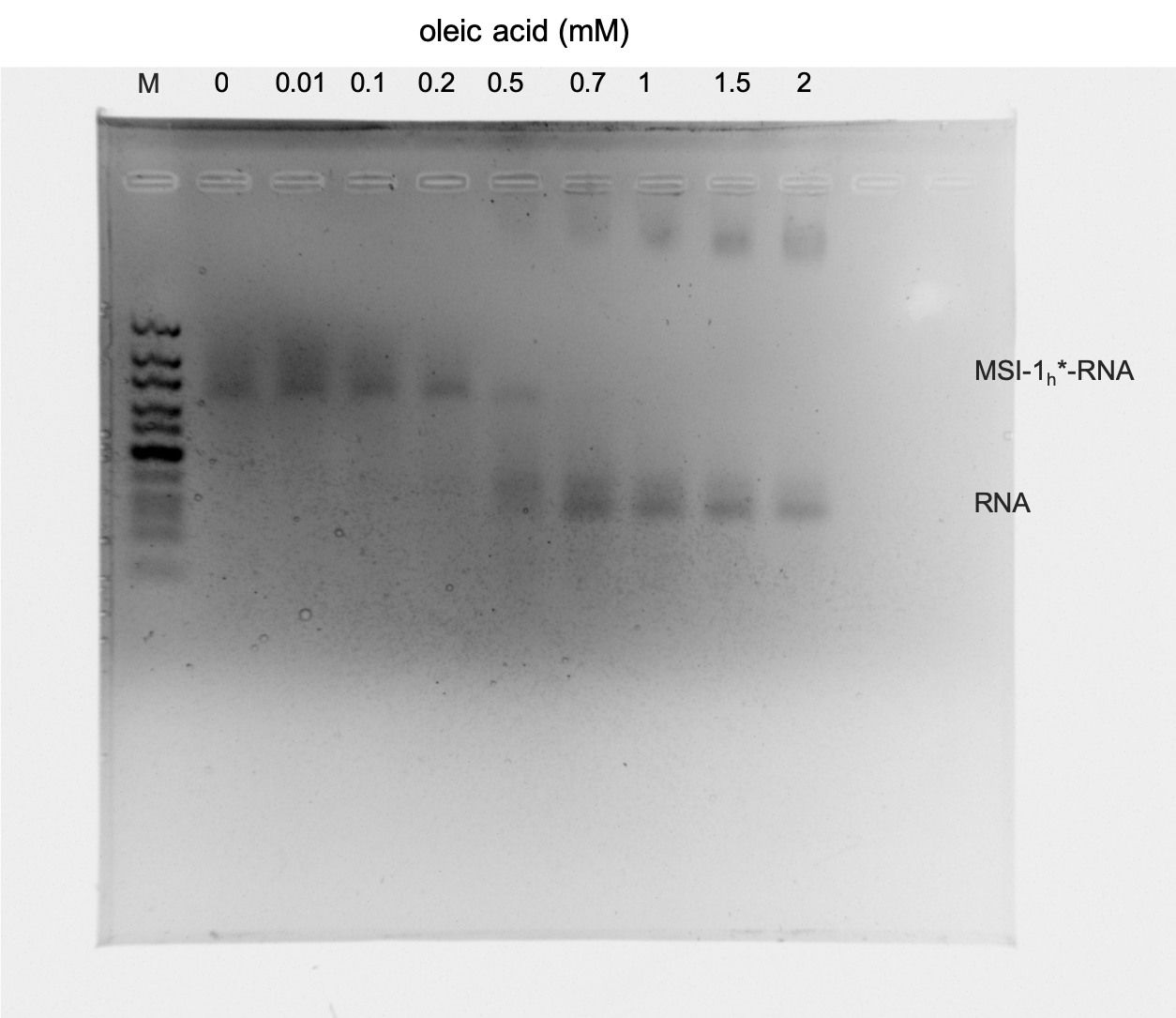

Supplement: Figure 5—figure supplement 1—source data 1. [file elife-91777-fig5-figsupp1-data1.zip › gel_fig5-figsuppl1b_labels.png]
